# Glucosamine Ameliorates Symptoms of High-Fat Diet-Fed Mice by Reversing Imbalanced Gut Microbiota

**DOI:** 10.3389/fphar.2021.694107

**Published:** 2021-06-03

**Authors:** Xubing Yuan, Junping Zheng, Lishi Ren, Siming Jiao, Cui Feng, Yuguang Du, Hongtao Liu

**Affiliations:** ^1^ State Key Laboratory of Biochemical Engineering and Key Laboratory of Biopharmaceutical Production and Formulation Engineering, PLA, Institute of Process Engineering, Chinese Academy of Sciences, Beijing, China; ^2^ Institute of Process Engineering, University of Chinese Academy of Sciences, Beijing, China

**Keywords:** glucosamine, gut microbiota, diabetes mellitus, inflammation, high fat diet

## Abstract

Glucosamine (GlcN) is used as a supplement for arthritis and joint pain and has been proved to have effects on inflammation, cancer, and cardiovascular diseases. However, there are limited studies on the regulatory mechanism of GlcN against glucose and lipid metabolism disorder. In this study, we treated high-fat diet (HFD)-induced diabetic mice with GlcN (1 mg/ml, in drinking water) for five months. The results show that GlcN significantly reduced the fasting blood glucose of HFD-fed mice and improved glucose tolerance. The feces of intestinal contents in mice were analyzed using 16s rDNA sequencing. It was indicated that GlcN reversed the imbalanced gut microbiota in HFD-fed mice. Based on the PICRUSt assay, the signaling pathways of glucolipid metabolism and biosynthesis were changed in mice with HFD feeding. By quantitative real-time PCR (qPCR) and hematoxylin and eosin (H&E) staining, it was demonstrated that GlcN not only inhibited the inflammatory responses of colon and white adipose tissues, but also improved the intestinal barrier damage of HFD-fed mice. Finally, the correlation analysis suggests the most significantly changed intestinal bacteria were positively or negatively related to the occurrence of inflammation in the colon and fat tissues of HFD-fed mice. In summary, our studies provide a theoretical basis for the potential application of GlcN to glucolipid metabolism disorder through the regulation of gut microbiota.

## Introduction

Diabetes mellitus is a chronic and systemic inflammatory disease characterized by hyperglycemia, which mainly includes three types: type 1 diabetes mellitus (T1DM) with insufficient insulin secretion, type 2 diabetes mellitus (T2DM) with insulin resistance, and gestational diabetes mellitus with raised blood glucose during pregnancy (Menini et al., 2020). The population of diabetic patients worldwide has reached 463 million as of 2019 and is expected to reach 700 million in 2045 (Belma et al., 2019). Among them, T2DM is more common in adults and accounts for up to 90% of patients (Zhang et al., 2020). It may be associated with an unhealthy diet, genetic inheritance, and environmental factors (Toi et al., 2020). In addition, a series of publications have demonstrated that gut microbiota is closely related to the occurrence and development of T2DM by direct (microorganisms themselves) or indirect (structural components or metabolites of microorganisms) interaction with the host (Wang et al., 2018; Herrema and Niess, 2020). Therefore, it is of significance to investigate the regulatory effect of gut microbiota on the prevention and treatment of T2DM.

Glucosamine (GlcN) is a naturally occurring amino sugar in the human body, and its salts include glucosamine hydrochloride and glucosamine sulfate, which are widely used in dietary supplements against osteoarthritis and joint pain (Reginster et al., 2001; Veronese et al., 2020). Studies show that GlcN has favorable biological activities, such as anti-inflammation, anti-bacteria, anti-cancer, and cardioprotection. And most of these bio functions are achieved by the regulation of inflammatory responses, especially the inhibition of Nuclear Factor-κB (NF-κB) signaling, inflammatory cytokine production, and enzymatic expression (Dalirfardouei et al., 2016). New evidence indicates that GlcN can initiate a positive regulatory effect on glucose metabolism disorder. In an average of 8.1 years of follow-up of 404,508 subjects, the habitual use of GlcN was closely related to the incidence of low-risk T2DM (Ma et al., 2020). In another clinical trial, the patients taking glucosamine sulfate orally for 3 years were accompanied by slightly decreased fasting blood glucose (Reginster et al., 2001). There were also reports in which the oral administration of glucosamine sulfate caused the abundance changes of gut microbiota (Shmagel et al., 2019). Based on the above, we hypothesize that GlcN may have a beneficial effect on diabetes mellitus through the regulation of gut microbiota.

In this study, we aim to determine the improvement of GlcN on glucose metabolism disorder in diabetic mice with high-fat diet (HFD) treatment. Also, by 16S rDNA sequencing, we investigated the role of gut microbiota in the amelioration of diabetic symptoms by GlcN.

## Materials and Methods

### Materials and Reagents

GlcN, i.e., D- (+)-Glucosamine hydrochloride was purchased from Sigma (St. Louis, MO, United States), and the structure was shown in Supplementary Figure S1 with the purity ≥99%. Hematoxylin and eosin staining kit was obtained from Beyotime Institute of Biotechnology (Jiangsu, China). Fluorescent secondary antibodies and 4′, 6-diamidino-2-phenylindole (DAPI) were purchased from Thermo Fisher Scientific (Waltham, MA, United States). The primers for Quantitative RT-PCR were synthesized by BGI Gene (Shenzhen, China). Other chemical reagents were from Beijing Chemical Company (Beijing, China).

### Animal Treatment

Twenty C57BL/six male mice (4–6 weeks old, 20 ± 2 g) were from the Model Animal Research Center of Nanjing University (Jiangsu, China). All mice were kept at a constant temperature (23 ± 2°C) and humidity with a 12 h light-dark cycle. During the one-week adaptation, mice were given a normal chow diet and drinking water ad libitum. After that, the mice were randomly divided into four groups (*n* = 5): 1) for normal chow diet (NCD) group, mice were fed with NCD (10 kcal% fat, D12450B); 2) for high-fat diet (HFD) group, mice were fed with HFD (45 kcal% fat; D12451); 3) for NCD + GlcN group, mice were fed with NCD and GlcN (1 mg/ml in drinking water, about 200 mg/kg/d); 4) for HFD + GlcN group, mice were fed with HFD and GlcN. Both NCD and HFD were purchased from KeAo Xieli Co., Ltd. (Beijing, China). To ensure the activity of GlcN, its aqueous solution was replaced every other day.

All the mice were raised in groups according to their different diets and drinking water for five months. During the GlcN treatment, the body weight, diet, and water drinking of mice were monitored.

### Intraperitoneal Glucose Tolerance Test

All mice were fasted for 12 h and then intraperitoneally injected with glucose (1 g/kg Body weight, dissolved in physiological saline). The tail vein blood was collected for blood glucose test by using an Accu-chek glucometer (Roche Diagnostics, Basel, Switzerland) at the time points of 0, 15 min, 30 min, 1 h, and 2 h after glucose injection.

### Organ Weight and Hematological Index

After the experiment, all mice were euthanized and dissected. Major tissues were collected and weighed, including heart, spleen, pancreas, brown adipose tissue (BAT), liver, white adipose tissue (WAT), and intestine. A part of WAT and colon tissues were fixed in 4% paraformaldehyde. The blood samples were collected for hematological index analysis (Nihon Kohden, Tokyo, Japan), and the fecal samples were collected for 16S rDNA sequencing. All samples were stored at −80°C for further experiment.

### Hematoxylin and Eosin Staining

The colon and WAT samples were fixed in 4% paraformaldehyde for 24 h followed by the embedding and then sliced (50 μm thickness). The paraffin sections were sequentially subjected to xylene dewaxing, gradient ethanol hydration, and H&E staining. The images were captured using a Leica DFC310 FX digital camera connected to a Leica DMI4000B light microscope (Wetzlar, Germany).

### RNA Extraction and Quantitative Real Time-Polymerase Chain Reaction

The colon and adipose tissues were homogenized using Trizon (CoWin Biosciences, Beijing, China) and homogenized. Then, chloroform, isopropanol, and ethanol were added in sequence for the extraction of total RNA. The concentrations and purity of extracted RNA were assayed with Q5000 ultra-micro nucleic acid protein analyzer (QUAWELL Technology Inc., United States). Reverse transcriptions were performed using 500 ng of high-quality total RNA with HiFiScript cDNA Synthesis Kit (CoWin Biosciences, Beijing, China). Quantitative RT-PCR (qRT-PCR) was performed with UltraSYBR Mixture High ROX Kit (CoWin Biosciences, Beijing, China) on a 7500 Fast Real-Time PCR System (Applied Biosystems, Foster City, CA, United States). The primer sequences used in this study were listed in Supplementary Table S1, including chemoattractant protein 1 (MCP-1), Interleukin 1β (IL-1β), Interleukin six (IL-6), Peroxisome proliferator-activated receptor gamma (PPARγ), and Integrin subunit alpha X (CD11c). Amplification protocol for 40 cycles was as follows: 2 min at 95°C for initial activation, 15 s at 95°C for denaturation, 60 s at 60°C for annealing/extension. Finally, β-Actin was used as a reference gene for the calculation of relative target gene expression using the 2^−ΔΔCT^ method.

### Detection of Gut Microbial Community

After mice were dissected, the fecal contents were collected, and the FastDNA™ SPIN Kit (MP Biomedicals, CA, United States) was used for genome extraction. Subsequently, 1% agarose gel electrophoresis and spectrophotometry detection at 260 nm/280 nm were conducted to detect the purity of extracted genomic DNA. Further, the extracted genomic DNA was used as a template. The amplification of 16S rRNA V3-V4 gene region was carried out using the 16S V3 338F forward (5′-ACT​CCT​ACG​GGA​GGC​AGC​AG-3′) and V4 806R reverse primers (5′-GGACTACHVGGGTWTCTAAT-3′) with Barcode. To ensure the accuracy and reliability of subsequent data analysis, low cycle number amplification (25 cycles) was used with the same cycles for each sample. Three replicates were performed, and the PCR products were detected by 2% agarose gel electrophoresis. The AxyPrepDNA gel recovery kit (Axygen Biosciences, CA, United States) was used to cut the gel for the recovery of PCR products.

The purified amplicon was quantified with QuantiFluor™-ST (Promega, United States) and adjusted to the same concentration for each sample. According to standard procedures, the Miseq library was constructed and the Miseq PE 300 second-generation high-throughput sequencing platform was used for paired-end (2 × 300) sequencing (Allwegene Technology Inc., Beijing, China). The obtained paired-end sequence data were used for filtering processing and data accusation. Subsequent data analysis and comparison were performed by using Quantitative Insights Into microbial Ecology (QIIME) software package, R language and Phylogenetic Investigation of Communities by Reconstruction of Unobserved States (PICRUSt) algorithm and platform.

### Statistical Analysis

Data were expressed as means ± SEM (Standard Error of Mean) and analyzed with Graphpad Prism 6 software (GraphPad Software, Inc., United States). The difference comparison and statistical analysis between groups were performed by a two-way ANOVA with Tukey-Kramer test. The LEfSe analysis on gut microbiota abundance and composition was based on Kruskal-Wallis and Wilcoxon tests, and the linear discriminant analysis (LDA) score threshold was 4.0–5.0. The comparison in Kyoto Encyclopedia of Genes and Genomes (KEGG) pathway was drawn with STAMP software. Statistical significance was considered at a *p*-value < 0.05.

## Results

### Effect of Glucosamine on Physiological Indices of High-Fat Diet-Fed Mice

**FIGURE 1:**
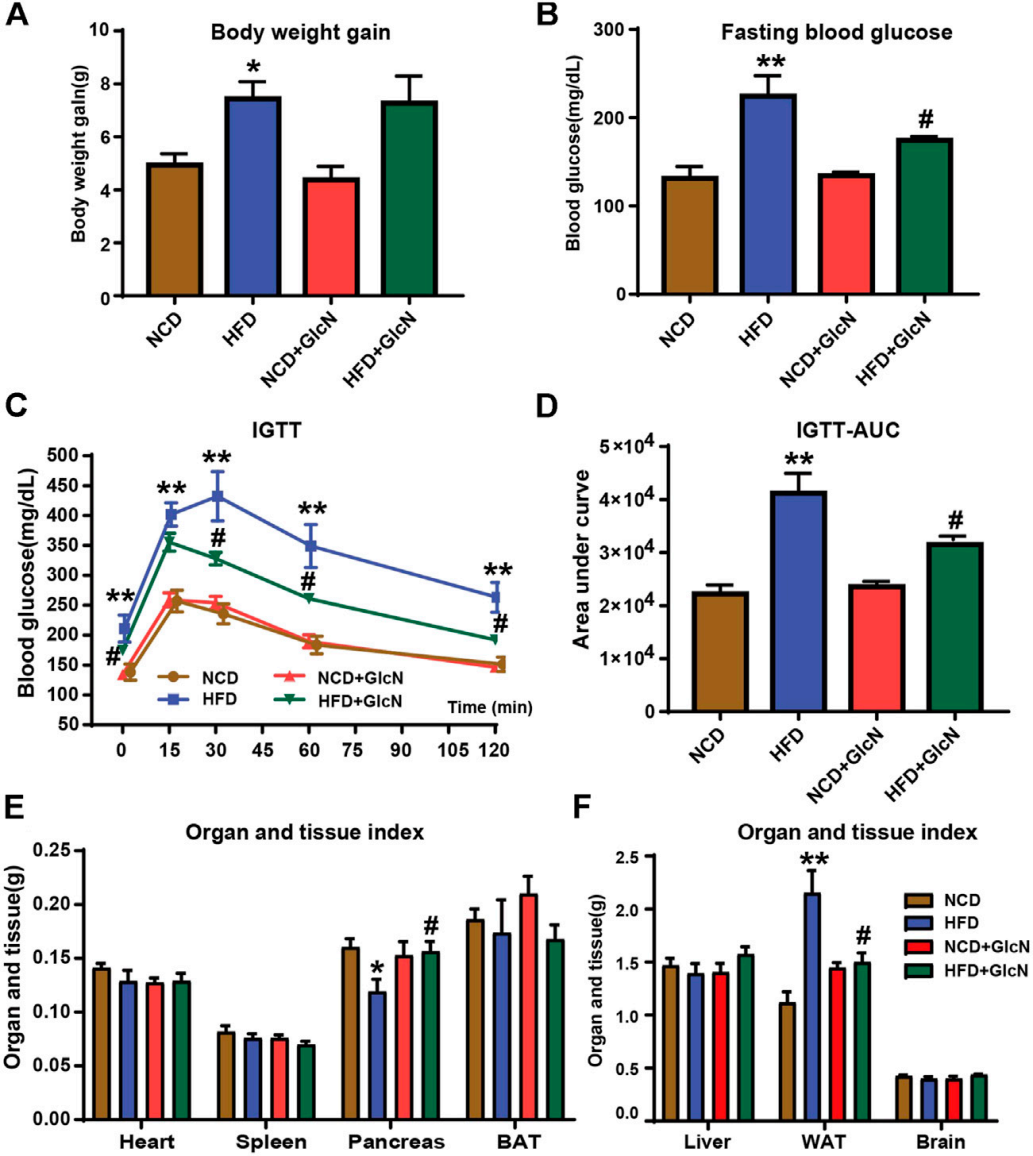
Effect of GlcN on pathophysiologic symptoms of diabetic mice induced by HFD-fed. After the mice were treated with NCD, HFD, and/or GlcN (1 mg/ml, in drinking water) for 5 months, the body weight **(A)** and fasting blood glucose **(B)** of mice were monitored. For the IGTT experiment, the blood glucose at different time points among experimental groups was tested **(C)**, and the area under the glucose tolerance curve was calculated **(D)**. Also, the visceral indices of major organs were detected, including heart, spleen, pancreas, BAT, liver, WAT, and brain **(E,F)**. Data were shown as means ± SEM (*n* = 5). *p < 0.05, **p < 0.01 indicates the difference between HFD group and NCD group; ^#^ *p* < 0.05, ^##^ *p* < 0.01 indicates the difference between HFD + GlcN group and HFD group.

After GlcN treatment for five months, the body weight and fasting blood glucose of mice in the HFD group were significantly higher than those in the NCD group (*p* < 0.05) (Figures 1A,B), proving that the diabetic mouse model was successfully constructed. Noticeably, GlcN treatment significantly reduced the blood glucose of HFD-fed mice (*p* < 0.05) (Figure 1B), indicating the improvement of GlcN on hyperglycemia in diabetic mice. As compared with the mice of the NCD group, the intolerance to glucose in the HFD group was proved by IGTT experiment, which was abrogated after GlcN treatment (Figure 1C). This was further confirmed by integrating the area under the curves of glucose tolerance among groups, which was significantly decreased by GlcN treatment (*p* < 0.05, vs. HFD group) (Figure 1D).

The mouse organ weight usually reflects the changes in their physiological conditions. Therefore, the heart, spleen, pancreas, BAT, liver, WAT, and brain of mice among experimental groups were weighed, and the visceral indices were calculated. It was found that the decrease in pancreatic weight and the increase in white adipose tissue weight in HFD-fed mice were statistically reversed by GlcN treatment (*p* < 0.05) (Figures 1E,F). It was indicated that GlcN improved the metabolic disorder of diabetic mice and especially suppressed the body's fat production and islet damage.

### Sequencing Data Validation and Alpha Diversity Analysis of Gut Microbiota

**FIGURE 2:**
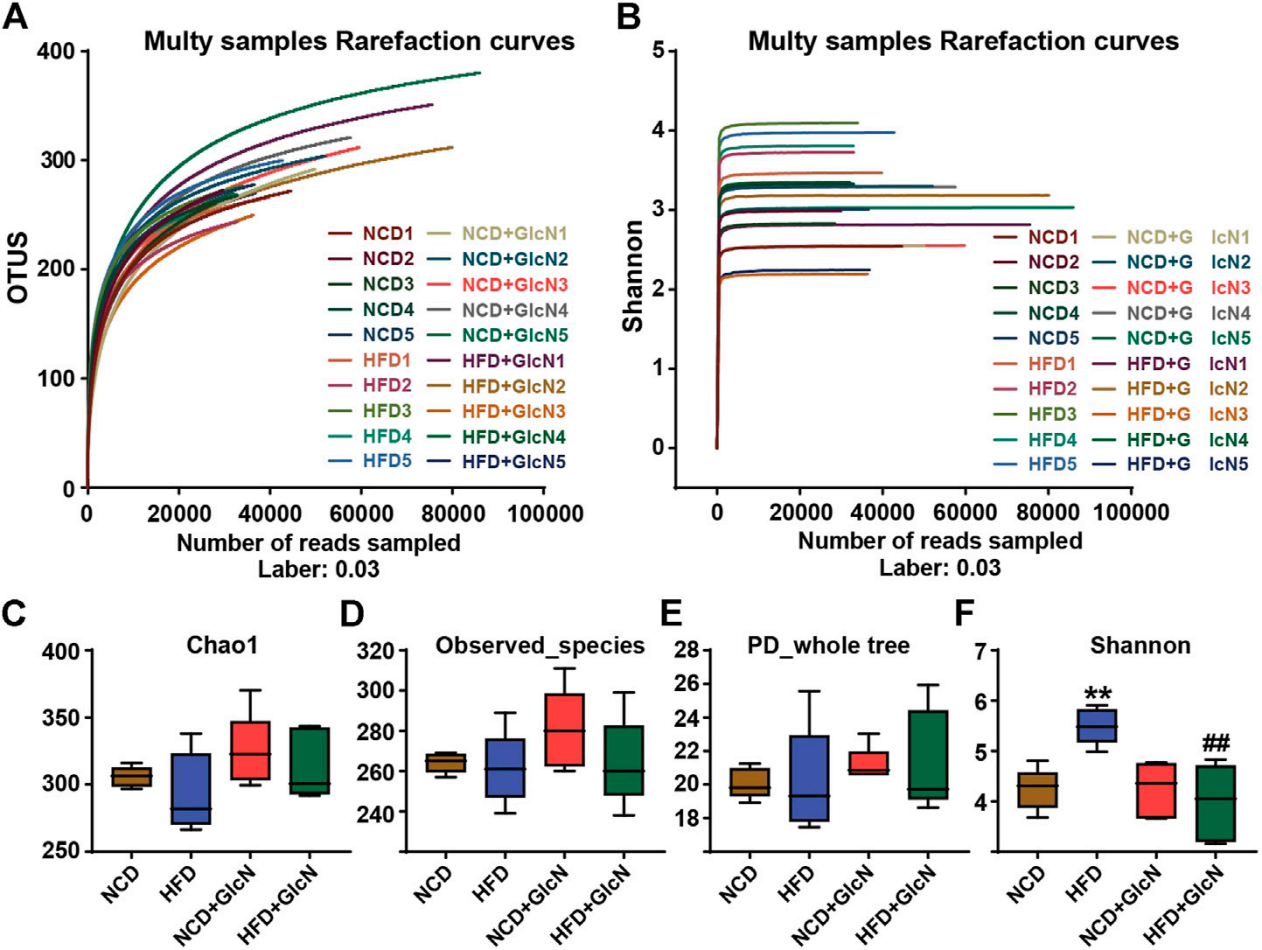
Sequencing data validation and diversity analysis of gut microbiota among experimental groups. After the treatment with NCD, HFD, and/or GlcN (1 mg/ml, in drinking water) for 5 months, all mice were sacrificed and the cecal contents were collected for 16S rDNA sequencing. Rarefaction curve **(A)** and Shannon-Wiener curve **(B)** were drawn by the OTU number sampled. Indices of Chao1 **(C)**, observed_species **(D)**, PD_whole_tree **(E)**, and Shannon index **(F)** were used for the α diversity analysis. Data were shown as means ± SEM (*n* = 5). **p < 0.01 indicates the difference between HFD group and NCD group; ^##^ *p* < 0.01 indicates the difference between HFD + GlcN group and HFD group.

The V3-V4 variable region sequences of gut microbiota in mice are classified according to their similarities, one of which is the Operational Taxonomic Units (OTU). By random sampling of these sequences, the Rarefaction Curve was constructed based on the number of reads sampled and the corresponding OTUs they represented (Figure 2A). The Shannon-Wiener Curve was presented by the microbial diversity index of each sample at different sequencing depths (Figure 2B). Both the Rarefaction Curve and Shannon-Wiener Curve tended to be flat, indicating that the amount of sequencing data was large enough to reflect the vast majority of microbial information in these samples.

The analysis of α diversity in a single sample reflects the abundance and diversity of the microbial community. Specifically, the indices of Chao1 (Figure 2C), observed_species (Figure 2D), and PD_whole_tree (Figure 2E) separately represent the species richness, number of observed OTUs, and pedigree diversity. The results show that there were no significant differences between the four experimental groups. As shown in Figure 2F, the Shannon index was significantly increased in the HFD group (*p* < 0.01, vs. NCD group), which was reduced by GlcN treatment (*p* < 0.01, vs. NCD group). It was suggested that GlcN treatment remarkably reversed the increase in intestinal bacteria diversity in HFD-fed mice.

### Analysis on Gut Microbiota Composition Among Experimental Groups

**FIGURE 3:**
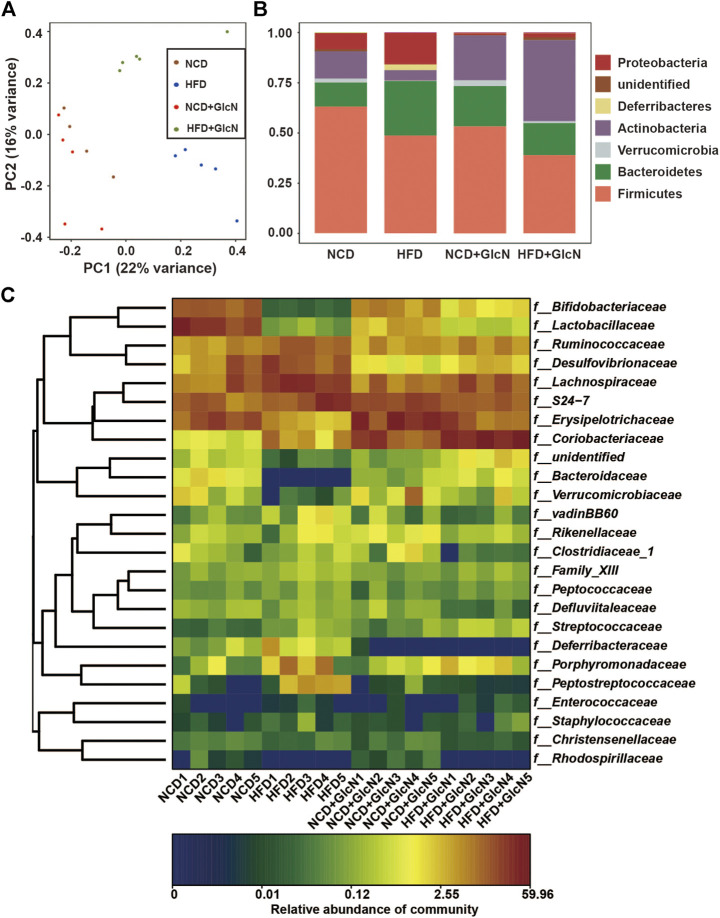
Effect of GlcN treatment on gut microbiota composition of HFD-fed mice. After the treatment with NCD, HFD, and/or GlcN (1 mg/ml, in drinking water) for 5 months, all mice were sacrificed and the cecal contents were collected for 16S rDNA sequencing. The differences in gut microbial diversity and abundance among four experimental groups were shown by Principal Component Analysis (PCA) **(A)**, histogram of phylum level **(B**), and heatmap of genus level **(C)**.

Principal Component Analysis (PCA), based on the evolutionary distance between species, is usually used to reflect the differences of microbial community composition among biological samples. The more similar the sample composition, the closer the distance that was reflected in PCA. As shown in Figure 3A, the OTUs between four experimental groups were distinguished, indicating the significant changes in compositions of gut microbiota.

In addition, as shown in Figure 3B, the abundances of major phyla in the gut of HFD-fed mice were significantly changed. Among them, Firmicutes and Actinobacteria were statistically reduced (*p* < 0.05, vs. NCD group), whereas Bacteroidetes, Proteobacteria, and Deferribacteres were markedly increased (*p* < 0.05, vs. the NCD group). In contrast, GlcN treatment significantly reversed the changes of Actinobacteria and Proteobacteria in the HFD-fed mice (*p* < 0.05). Also, the abundances of several undefined microbes were found to be affected by HFD feeding (*p* < 0.05, vs. NCD group). More specifically, as shown in Figure 3C, there are significant changes in the composition and abundance of intestinal microbes between different experimental groups at the family level.

**FIGURE 4:**
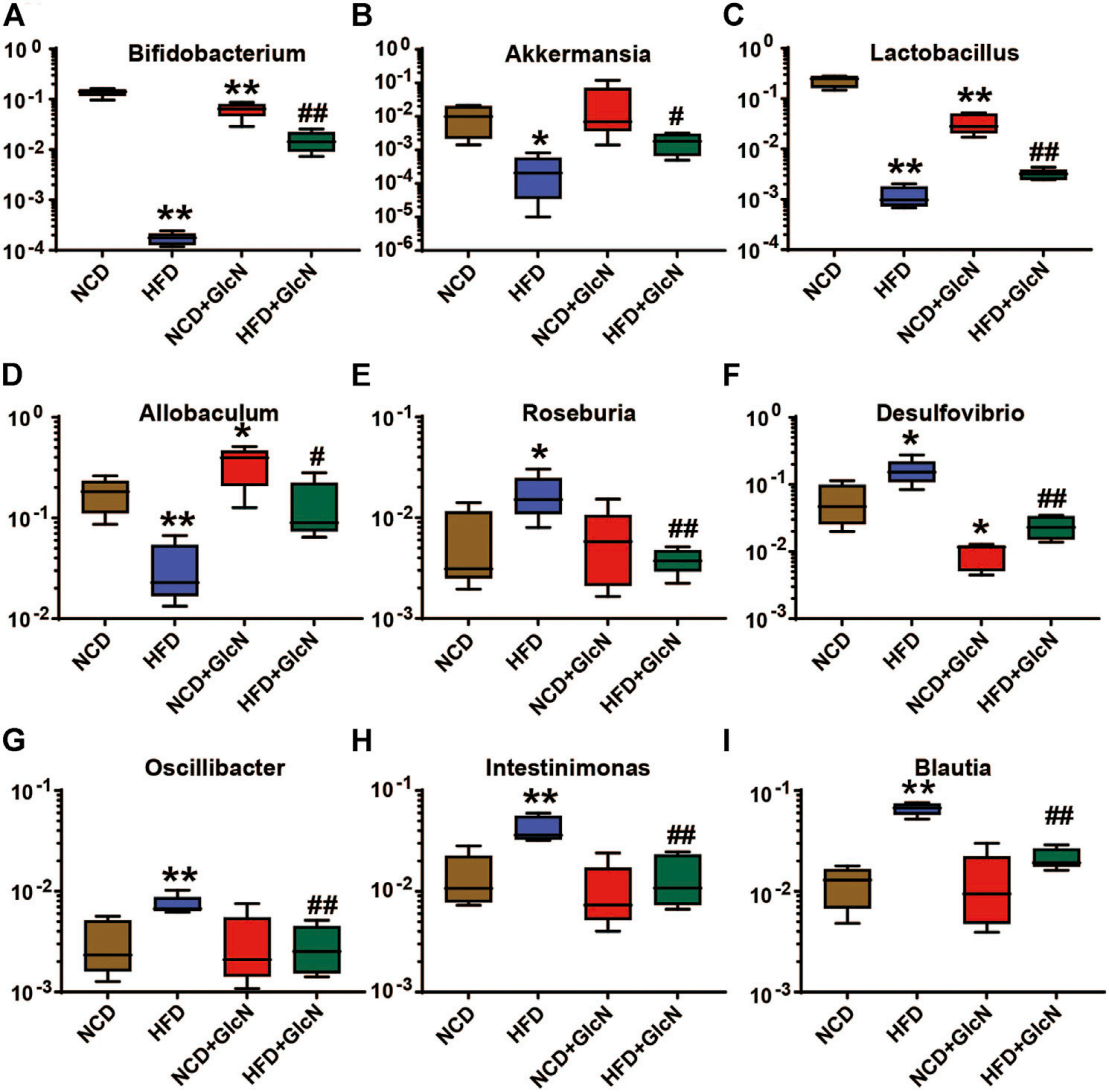
Effect of GlcN treatment on relative abundances of gut microbiota in HFD-fed mice at the genus level, including *Bifidobacterium* **(A)**, *Akkermansia* **(B)**, *Lactobacillus* **(C)**, *Allobaculum* **(D)**, *Roseburia* **(E)**, *Desulfovibrio* **(F)**, *Oscillibacter* **(G)**, *Intestinimonas* **(H)**, and *Blautia* **(I)**. Data were shown as means ± SEM (*n* = 5). *p < 0.05, **p < 0.01 indicates the difference between HFD group and NCD group; ^#^ *p* < 0.05, ^##^ *p* < 0.01 indicates the difference between HFD + GlcN group and HFD group.

To further clarify the effect of GlcN treatment on the composition structure of specific bacteria, we analyzed the population changes of intestinal microbes at the genus level among four experimental groups. As shown in Figure 4, the abundances of several beneficial bacteria in the HFD group were significantly lower than those in the NCD group, including *Bifidobacterium* (*p* < 0.01) (Figure 4A), *Akkermansia* (*p* < 0.05) (Figure 4B), *Lactobacillus* (*p* < 0.01) (Figure 4C), and *Allobaculum* (*p* < 0.01) (Figure 4D). After the treatment with GlcN, the contents of above-mentioned bacteria were significantly promoted (*p* < 0.05). Besides, GlcN treatment reversed the increase of some harmful bacteria in the gut microbes of HFD-fed mice, including *Roseburia* (Figure 4E) (*p* < 0.01), *Desulfovibrio* (Figure 4F) (*p* < 0.01), *Oscillibacter* (Figure 4G) (*p* < 0.01), *Intestinimonas* (Figure 4H) (*p* < 0.01), and *Blautia* (Figure 4I) (*p* < 0.01). In general, GlcN regulated the imbalance of gut microbes caused by HFD feeding.

### Bacteria Contributed to Gut Microbiota Variety Among Experimental Groups

**FIGURE 5:**
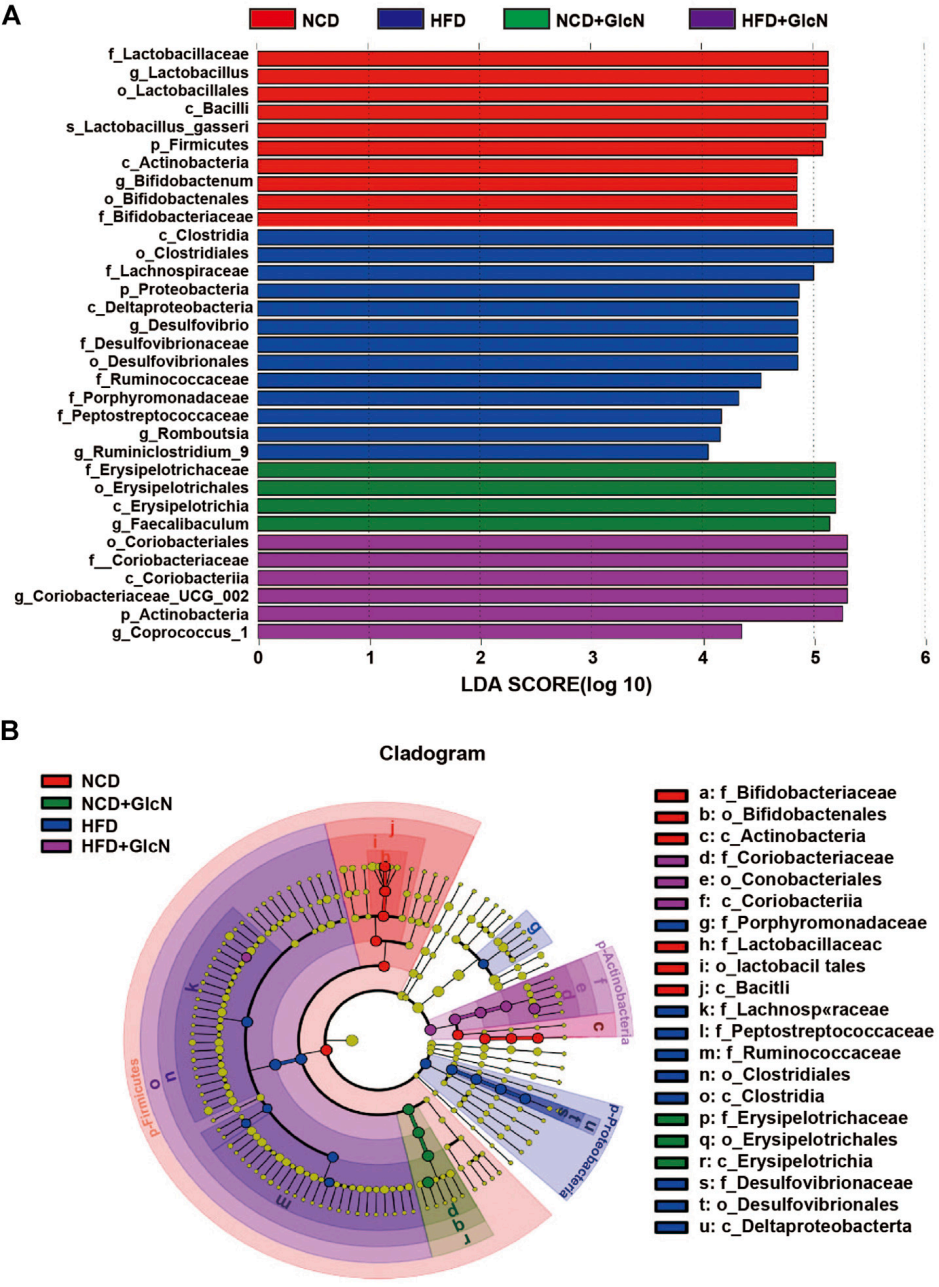
Identification of most significantly contributed intestinal microbes among experimental groups using Linear discriminant analysis (LDA) and Linear discriminant analysis Effect Size (LEfSe). After the treatment with NCD, HFD, and/or GlcN (1 mg/ml, in drinking water) for 5 months, all mice were sacrificed and the cecal contents were collected for 16S rDNA sequencing. LEfSe analysis was performed based on the intestinal microbial abundances and compositions, and the results were displayed in the form of LDA value distribution histogram **(A)** and cladogram **(B)**.

Through linear discriminant analysis effect size (LEfSe) analysis, we identified the representative gut microbes in each experimental group (Figure 5). Based on the LDA scores (Figure 5A) and cladogram assay (Figure 5B), the dominant gut microbes in the HFD group include Lachnospiraceae, Desulfovibrionaceae, Porphyromonadaceae, Ruminococcaceae, and Peptostreptococcaceae at the family level, and *Desulfovibrio*, *Romboutsia*, and *Ruminiclostridium_9* at the genus level. In contrast, the most significant gut microbes in the HFD + GlcN group were f_ Coriobacteriaceae and the corresponding *Coriobacteriacea_UCG_002* and *Coprococcus_1* at the genus level, showing the effect of GlcN treatment on the two types of microbes in HFD-fed mice.

### Effect of Glucosamine on Metabolic Function of Gut Microbiota in High-Fat Diet-Fed Mice

**FIGURE 6:**
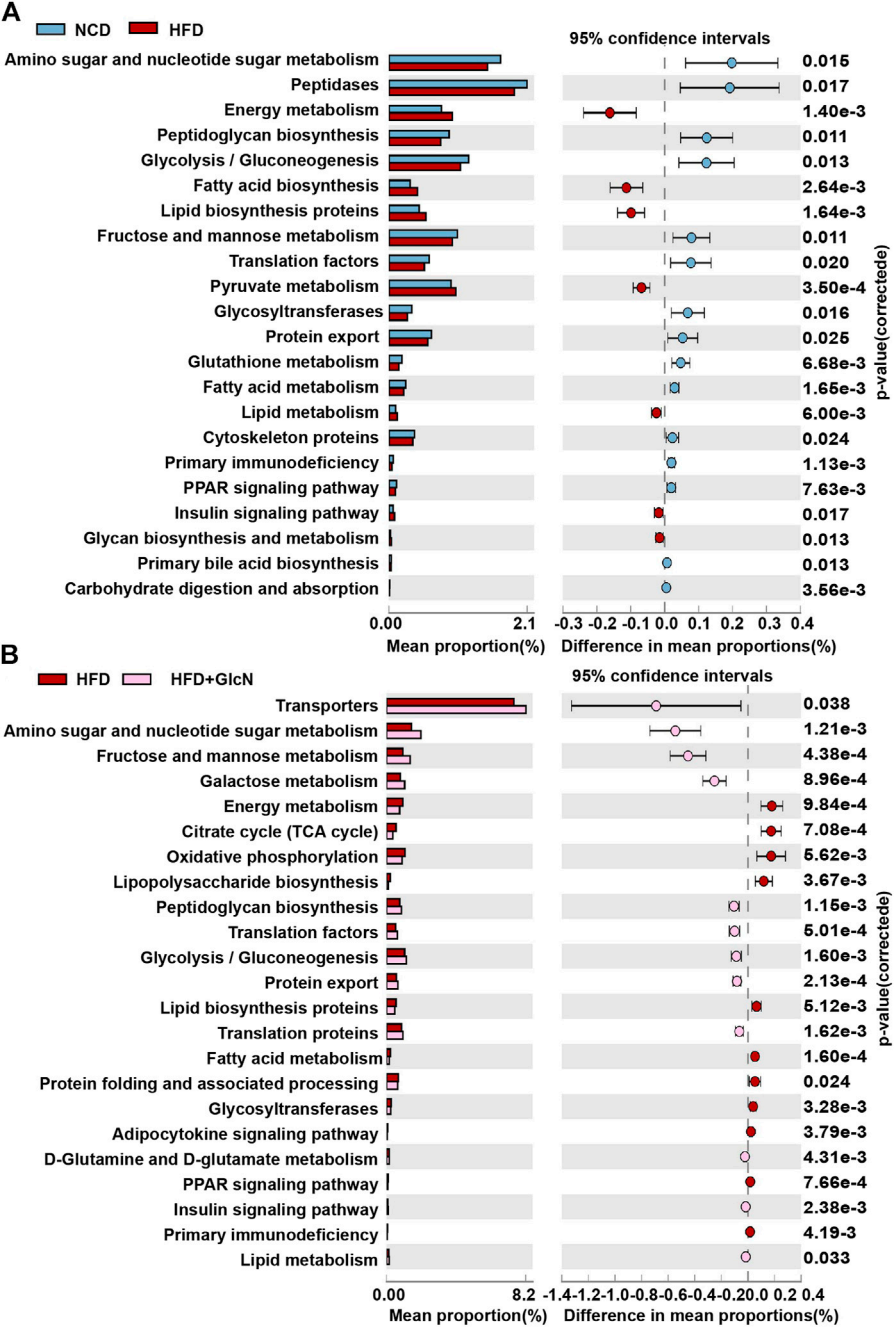
Metabolic function changes of gut microbiota among experimental groups. After the treatment with NCD, HFD, and/or GlcN (1 mg/ml, in drinking water) for 5 months, all mice were sacrificed and the cecal contents were collected for 16S rDNA sequencing. By using PICRUSt analysis, the metabolic alteration of KEGG pathways in the HFD group **(A)** and the reversal effect of GlcN treatment **(B)** were analyzed.

The change in gut microbiota composition often means the alteration of their bio functions. Thus, we explored metabolic pathways of the intestinal microbe community among groups by PICRUSt. As shown in Figure 6A, 22 KEGG pathways of HFD-fed mice were found to be changed as compared to those of mice in the NCD group. Among these pathways, glucolipid metabolism was the most involved ones, including amino sugar and nucleic acid sugar metabolism, energy metabolism, glycolysis/gluconeogenesis, fructose and mannose metabolism, pyruvate metabolism, glutathione metabolism, fatty acid metabolism, lipid metabolism, glycan synthesis and metabolism, carbohydrate digestion and absorption. Other changed pathways were mainly associated with biosynthesis and signaling transduction. After GlcN treatment, nine KEGG were improved compared with the HFD group such as amino sugar and nucleic acid sugar metabolism, fructose and mannose metabolism, and energy metabolism (Figure 6B).

### Suppressive Effect of Glucosamine on Damage to Colon Tissues of High-Fat Diet-Fed Mice

**FIGURE 7:**
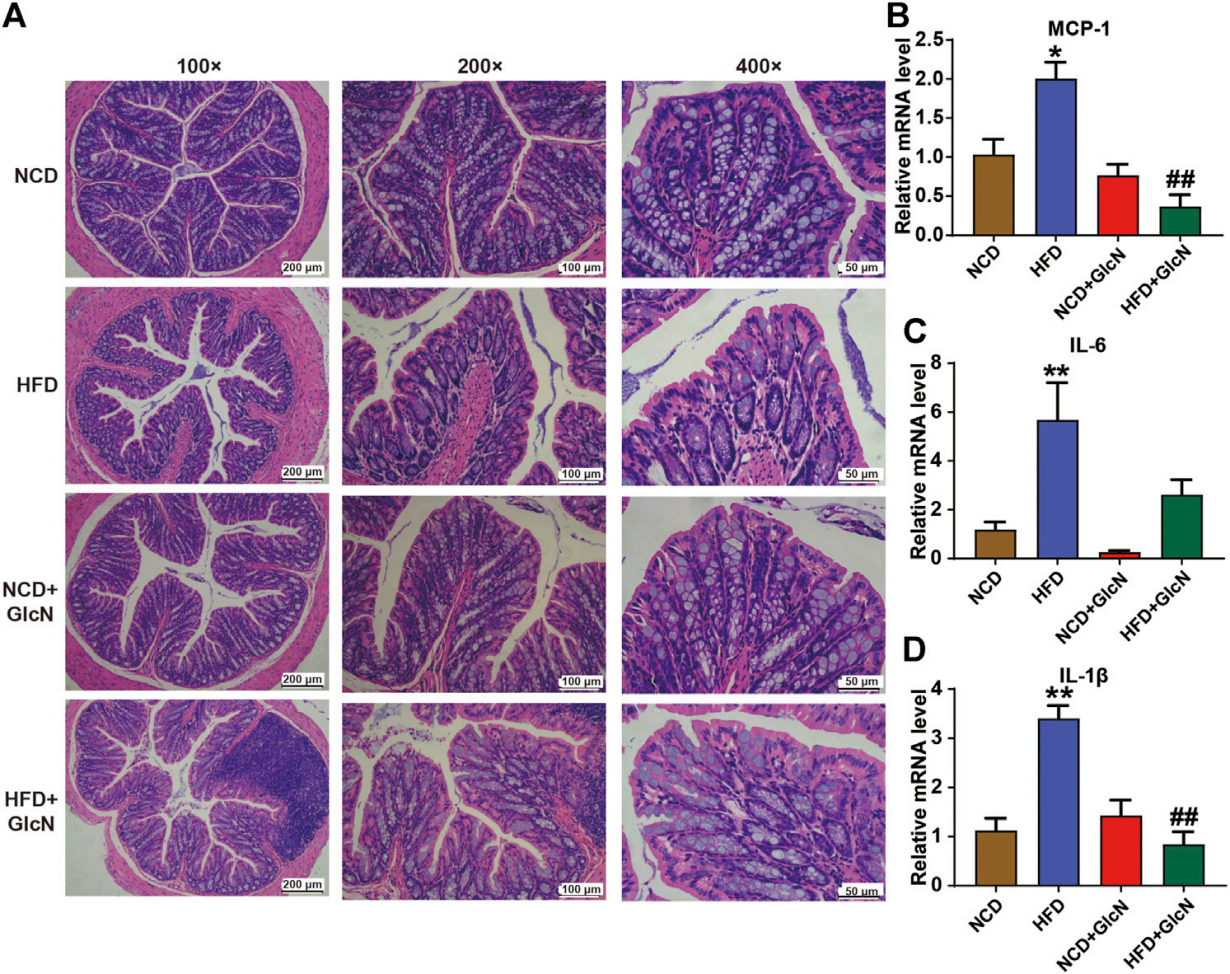
GlcN suppressed inflammatory responses in colon tissues of HFD-fed mice. After the treatment with NCD, HFD, and/or GlcN (1 mg/ml, in drinking water) for 5 months, all mice were sacrificed. The colon tissues were stained by H&E and photographed under a microscope (magnification of ×100, ×200 and ×400) **(A)**. The expressions of MCP-1 **(B)**, IL-6 **(C)**, and IL-1β **(D)** in colon at mRNA level were detected by qRT-PCR. Data were shown as means ± SEM (*n* = 5). *p < 0.05 indicates the difference between HFD group and NCD group; ^##^ *p* < 0.01 indicates the difference between HFD + GlcN group and HFD group.

Next, we investigated the protection of GlcN against HFD-induced damage to colon tissues in mice. As indicated in Figure 7A by H&E staining, the mucin of intestinal barrier of HFD-fed mice was significantly reduced, which was improved after GlcN treatment, indicating the protective effect of GlcN on the integrity of colon tissues in HFD-fed mice. Further experiment confirmed that GlcN treatment inhibited the mRNA expressions of MCP-1 (*p* < 0.01, vs. HFD group), IL-6 and, IL-1β (*p* < 0.01, vs. HFD group) to a large extent (Figures 7B–D).

### Inhibitory Effect of Glucosamine on Inflammation in White Adipose Tissues and Blood of High Fat Diet-Fed Mice

**FIGURE 8:**
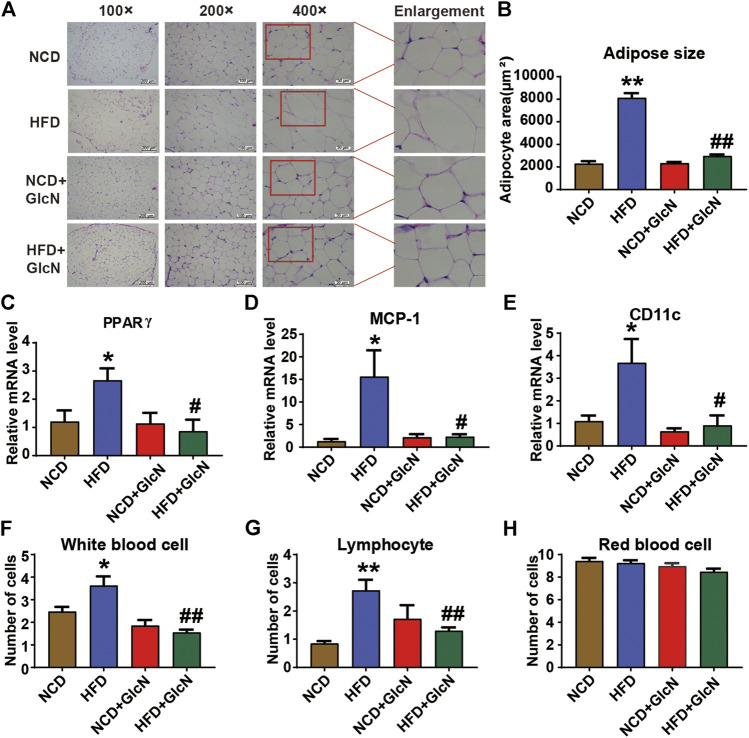
GlcN inhibited inflammatory responses in white adipose tissues (WATs) and blood of HFD-fed mice. After the treatment with NCD, HFD, and/or GlcN (1 mg/ml, in drinking water) for 5 months, all mice were sacrificed. WATs were morphologically observed by H&E staining **(A)** and the size of adipocytes was statistically analyzed **(B)**. The mRNA levels of pro-inflammatory and adipose differentiation-related cytokines were determined by RT-PCR, including PPARγ **(C)**, MCP-1 **(D)**, and CD11c **(E)**. The number of white blood cells **(F)**, lymphocytes **(G)**, and red blood cells **(H)** in the blood of each experimental group were also measured. Data were shown as means ± SEM (*n* = 5). *p < 0.05, **p < 0.01 indicates the difference between HFD group and NCD group; ^#^ *p* < 0.05, ^##^ *p* < 0.01 indicates the difference between HFD + GlcN group and HFD group.

WAT is involved in a variety of physiological or pathological processes such as insulin sensitivity, inflammation, and glucose and lipid metabolism. By H&E staining, it was observed that HFD-fed mice had severe adipose hyperplasia (Figure 8A), while GlcN treatment significantly reduced the adipocyte size of WATs (*p* < 0.01, vs. HFD group) (Figure 8B). To further confirm the beneficial effect of GlcN on WATs, the mRNA levels of several pro-inflammatory cytokines were measured by qRT-PCR. The result shows that the expressions of PPARγ (Figure 8C), MCP-1 (Figure 8D), and CD11c (Figure 8E) in the HFD grwhy usoup were significantly increased (*p* < 0.05, vs. NCD group), which were inhibited by GlcN (*p* < 0.05, vs. NCD group). Also, GlcN remarkably reduced the number of white blood cells (Figure 8F) and lymphocytes (Figure 8G) in the blood of HFD-fed mice. Besides, the treatment of GlcN and/or HFD had no effect on the number of red blood cells (Figure 8H).

### Correlation Analysis Between Gut Microbiota and Physiochemical Indices in High Fat Diet-Fed Mice

**FIGURE 9:**
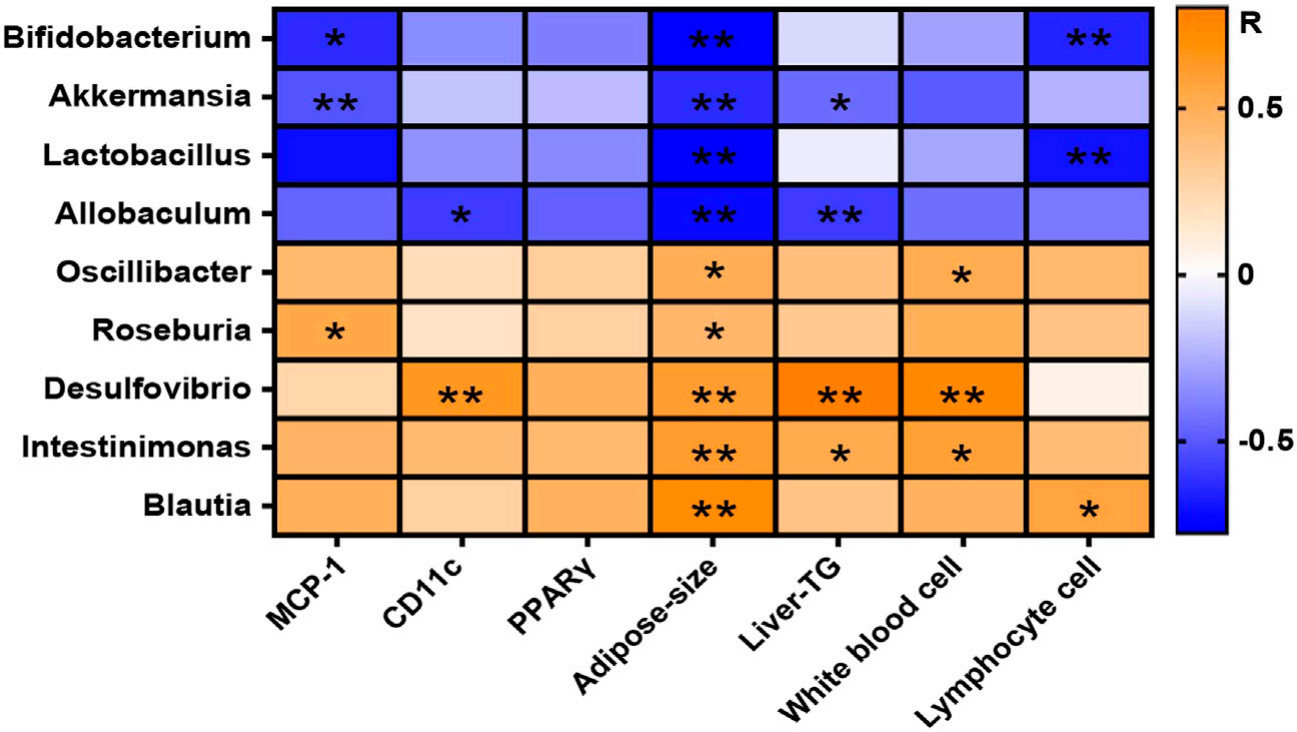
Correlation between gut microbiota and physiochemical indices among experimental groups. After the treatment with NCD, HFD, and/or GlcN (1 mg/ml, in drinking water) for 5 months, all mice were sacrificed and the cecal contents were collected for 16S rDNA sequencing. The correlation between changed gut microbiota at the genus level and physiochemical indices was displayed in a heat map. The orange-white-blue grid corresponded to the *R*-value of 0.5-0-(−0.5). Orange color, positive correlation; blue color, negative correlation. Data were shown as means ± SEM (*n* = 5). *p < 0.05, **p < 0.01.

The correlation of intestinal microbes changed significantly at the genus level and the representative physiological indicators of mice were analyzed by Spearman correlation and displayed in the form of a heat map (Figure 9). The result confirmed that some beneficial bacteria including *Bifidobacterium*, *Akkermansia*, *Lactobacillus*, and *Allobaculum* were negatively correlated with the level of MCP-1 in WAT, the adipose size, the lymphocyte number, and the liver triglyceride (Liver-TG) content in blood. Other bacteria with reduced abundances after GlcN treatment, including *Oscillibacter*, *Roseburia*, *Desulfovibrio*, *Intestinimonas*, and *Blautia*, were positively correlated with the levels of MCP-1 and CD11c in WAT, the adipose size, the numbers of white blood cells and lymphocytes, and the Liver-TG content in blood, implying that the changes of gut microbes affected the inflammatory responses of host.

## Discussion

**FIGURE 10:**
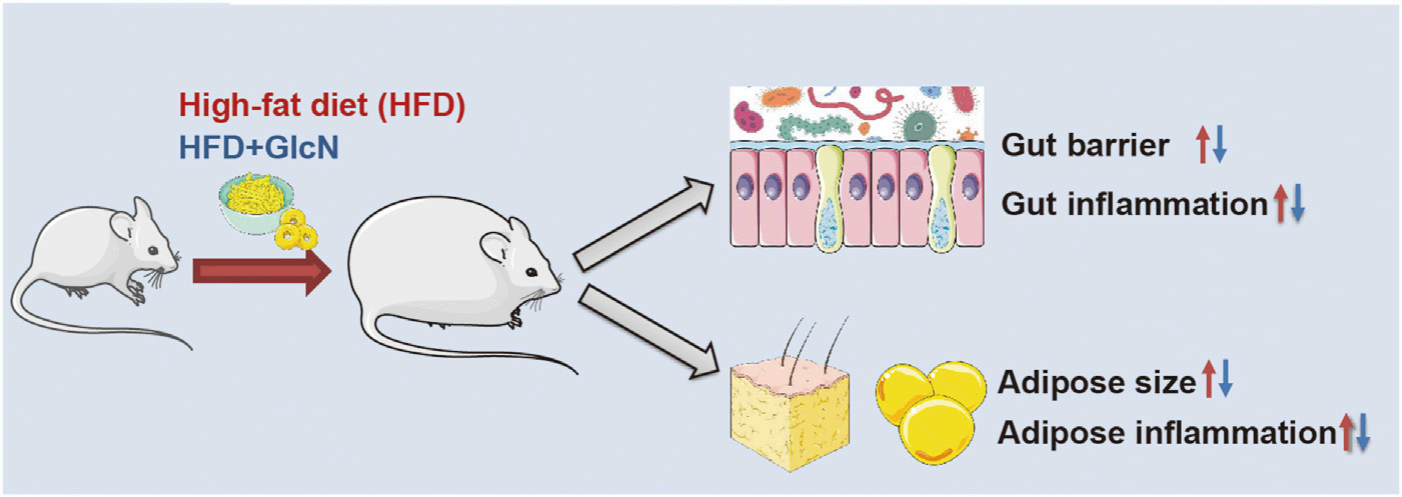
Graphical overview of possible mechanisms by which GlcN improved glucolipid metabolism disorder of HFD-fed mice.

In this study, we documented that in the diabetic model with dysregulated glucolipid metabolism established by HFD feeding, GlcN supplementation (1 mg/ml in drinking water) could reverse the imbalance of intestinal flora, intestinal barrier damage, and inflammatory responses of colon tissues and blood, accompanied by the improvement of physiological indices such as blood sugar and glucose tolerance in HFD-fed mice (Figure 10).

Obesity, as a chronic relapsed disease process, is an important driving force for diabetes and many other diseases (Johansson and Hansson, 2016; Menini et al., 2020; Murray et al., 2020). Among them, diet is the primary contributor, especially foods with high energy density such as high-fat and high-sugar diets (Bray et al., 2017). In this study, we adopted a high-fat diet of 45 kcal% fat to construct diabetic mouse model with glucose and lipid metabolism disorder. This method has been generally applied to metabolism-related pharmacological studies (Karim et al., 2018; Li et al., 2019). After the HFD-fed mice were intervened with GlcN in drinking water, we found that GlcN significantly reduced the fasting blood glucose and improved glucose tolerance of HFD-fed mice. In addition, GlcN decreased the weight of WATs of HFD-fed mice, suggesting the inhibitory effect on adipogenesis (Figure 1). This result was partly consistent with previous studies, in which GlcN improved insulin resistance and glucose intolerance caused by HFD, but increased the body weight and fat weight of normal mice (Hwang et al., 2015). This divergence may be due to the difference in physiological conditions of mice and GlcN dosage used.

In recent years, a line of evidences has demonstrated that diabetes is closely related to the imbalance of intestinal microbes (Arora et al., 2021). As early as 2007, it was reported that sterile mice were less likely to be obese than ordinary mice even if they were fed with HFD (Backhed et al., 2007). In contrast, germ-free mice receiving gut microbes from obese mice exhibited an obese phenotype, suggesting the involvement of intestinal floral in the occurrence of obesity (Clarke et al., 2014). Specifically, the ratio of Firmicutes to *Bacteroides* in mice fed with HFD was reduced by 50–60% (Yan et al., 2017). This result was in accordance with the phylum changes in our 16s sequencing data (Figure 3B). At the genus level, we found that GlcN could evidently restore the altered abundances of *Bifidobacterium*, *Akkermansia*, *Lactobacillus*, *Allobaculum*, *Roseburia*, *Desulfovibrio*, *Intestinimonas*, *Blautia*, and *Oscillibacter* in the gut of HFD-fed mice (Figure 4). Among them, *Bifidobacterium* and *Lactobacillus* were recognized as physiologically beneficial bacteria, which displayed positive effects such as regulating intestinal flora, enhancing immunity, improving intestinal function, and alleviating insulin resistance in diabetic mice (Hsieh et al., 2018). As a new generation of probiotics, the abundance of *Akkermansia* was reduced in obesity, diabetes mellitus and other diseases, followed by the destroyed intestinal barrier, increased plasma endotoxin level, and chronic inflammation (Everard et al., 2013). And the supplementation with *Akkermansia* could significantly prevent mice from obesity caused by HFD and reduced blood sugar in diabetic mice (Plovier et al., 2017; Depommier et al., 2019). In addition, *Allobaculum* was found to be the main reason for the suppression of glucose digestion in host, and its abundance was also reduced in HFD-fed mice (Everard et al., 2014; Herrmann et al., 2017). On the contrary, our results show that GlcN inhibited the growth of harmful bacteria in HFD-fed mice, including the pro-inflammatory bacteria *Desulfovibrio* and the alcoholic fatty liver-related bacteria *Blautia* (Lennon et al., 2014; Zhang-Sun et al., 2015; Shen et al., 2017). The results meant that GlcN could regulate the intestinal microbe imbalance in HFD-fed mice. As far as we know, there is no report about the regulatory effect of GlcN on intestinal microbes. Of course, rodents and humans have different diets, and there are also differences in the microbiota in the intestines, which is also our limitation. In subsequent studies, it is necessary to recruit volunteers to conduct human experiments to verify our results.

By using PICRUSt analysis to predict the functional changes of gut microbiota due to their altered compositions and abundances, we observed that GlcN mainly interfered with the glucolipid metabolism and biosynthesis pathways of intestinal floral in HFD-fed mice (Figures 6A,B). Among these pathways, glycolysis/gluconeogenesis, lipid biosynthesis protein, and energy metabolism are closely associated with the development of diabetes mellitus (Kosanam et al., 2014; Batista et al., 2021; Wang et al., 2021). As an effective arthritis inhibitor, GlcN has been proved to suppress the activation of nucleotide-binding oligomerization domain-like receptor containing pyrin domain 3 (NLRP3) inflammasome in mouse and human macrophages. After oral administration, it could down-regulate the concentrations of pro-inflammatory cytokines (IL-1β, IL-6, MCP-1, and TNF-α) and thus displayed anti-inflammatory activity (Chiu et al., 2019). Indeed, our study also documented that GlcN statistically abrogated the inflammatory attack in colon and fat tissues of HFD-fed mice (Figure 7; Figure 8). Based on previous reports, the damage to the intestinal barrier will not only cause the entrance of harmful bacteria into the blood, but also increase the endotoxin level in the circulatory system. These pathological stimuli lead to organ inflammation, *in vivo* oxidative stress, and fat accumulation in the development of diabetes mellitus. Since white adipose tissue, muscle, and liver are major tissues responsible for glucose and lipid metabolism, their damages by pathological intestinal bacteria or bacterial products will directly induce the dysfunction of glucose metabolism in the body. Considering that the imbalance of intestinal microbes will result in the destruction of the intestinal barrier and subsequent inflammatory responses leading to the disorder of glucose and lipid metabolism (Cani et al., 2007; Cani et al., 2008; Rouland et al., 2021), we speculate that GlcN may inhibit the inflammatory responses in HFD-fed mice by reversing the imbalance of gut microbiota. This was confirmed by the correlation analysis, in which the most significantly changed intestinal bacteria were positively or negatively related to the occurrence of inflammation in the colon and fat tissues of HFD-fed mice (Figure 9).

In conclusion, our studies proved that GlcN could regulate the composition and function of gut microbiota in HFD-fed mice, suppressed the inflammatory responses of colon and fat tissues, and thus improved physiological indices like glucose tolerance. Our results provide a theoretical basis for the potential application of GlcN to glucolipid metabolism disorder through the regulation of gut microbiota.

## Data Availability

The data presented in the study are deposited in the (online) repository, accession number (NCBI PRJNA731000).

## Supplementary Material

**SUPPLEMENTARY FIGURE 1** Structure diagram of glucosamine hydrochloride used in this study.

**SUPPLEMENTARY TABLE 1 **The primer sequence used for RT-qPCR.
