# Supplementary material for: Glucosamine Ameliorates Symptoms of High-Fat Diet-Fed Mice by Reversing Imbalanced Gut Microbiota
Source: Front Pharmacol. 2021 Jun 3;12:694107. doi: 10.3389/fphar.2021.694107 (PMC8209492; doi:10.3389/fphar.2021.694107)
Supplement: Supplementary file 1 [file DataSheet1.docx]

Supplementary Material

# Supplementary Figures and Tables

**Supplementary Figure 1.** Structure diagram of glucosamine hydrochloride used in this study.

**Supplementary Table 1.** The primer sequence used for RT-qPCR.

## Supplementary Figure


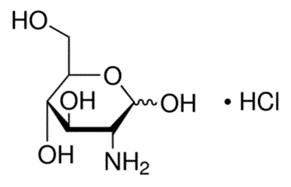


**Supplementary Figure 1.** Structure diagram of glucosamine hydrochloride used in this study.

## Supplementary Table

**Table 1.** The primer sequence used for RT-PCR

| Name | Forward primer | Reverse primer |
| --- | --- | --- |
| IL-1β | CGACAAAATACCTGTGGCCT | TTCTTTGGGTATTGCTTGGG |
| IL-6 | GAAACCGCTATGAAGTTCCTCTCTG | TGTTGGGAGTGGTATCCTCTGTGA |
| MCP-1 | GGGATCATCTTGCTGGTGAA | AGGTCCCTGTCATGCTTCTG |
| PPARγ | TCGCTGATGCACTGCCTATG | GAGAGGTCCACAGAGCTGATT |
| CD11c | ACGTCAGTACAAGGAGATGTTGGA | ATCCTATTGCAGAATGCTTCTTTACC |
| β-Actin | AGGTGACAGCATTGCTTCTG | GCTGCCTCAACACCTCAAC |
